# An Idealized Method of Simulating Residual Ionospheric Errors in Radio Occultation

**DOI:** 10.1038/s41598-017-16972-4

**Published:** 2017-11-30

**Authors:** Zhiqiang Fan, Zheng Sheng, Peng Guo, Hanqing Shi

**Affiliations:** 1 College of Meteorology and Oceanology, National University of Defense Technology, Nanjing, 211101 China; 2 Shanghai Astronomical Observatory, National Academy of Sciences, Shanghai, 200030 China

## Abstract

An idealized model is used to simulate radio occultation bending angles and residual ionospheric errors. The test results of the proposed simulation method agree with those of previous studies that use end-to-end simulation tools. Also, a new residual ionospheric error model proposed by Healy and Culverwell (2015) is verified in this letter by characterizing the key parameter, κ. A simple model, κ(*a*) = **A** − **B** × (*a* − 20)/60, is used to estimate the values of κ, where **A** and **B** are constants that indicate the magnitude and variation of the values of κ, respectively, and *a* represents the impact height. When the modelled values of κ are applied in performing ionospheric corrections, the residual ionospheric errors decrease from approximately 5 × 10^−8^ rad to 1 × 10^−9^ rad at a latitude of 40°N during the daytime and at a solar activity level of F_10.7_ = 210. Though the proposed model does not assess other error terms, such as those associated with asymmetry and noise, it will likely prove to be an effective tool for describing idealized residual ionospheric errors in radio occultation, and the features of the κ values identified in this study may be helpful in improving ionospheric correction methods.

## Introduction

The radio occultation (RO) technique is a popular measurement method that is used to observe the atmosphere, and its products are widely used in global climate monitoring and numerical weather prediction^1,2^. The ionosphere is commonly considered to be a major source of error in radio occultation measurements at heights above 30 km^3,4^. As height increases, the influence of the ionosphere becomes more dominant; thus, the errors in the radio occultation retrievals are significant^5^. Currently, a linear combination of dual-frequency bending angles proposed by Vorob’ev and Krasil’nikova (1994) is used to correct for ionospheric effects, and this method can remove the first-order ionospheric contributions^6^. However, the higher-order ionospheric residuals remain, and the remaining residual ionospheric errors (RIEs), which lead to significant bias in radio occultation retrievals, especially at heights above 30 km, are not negligible^7,8^.

The residual ionospheric errors in bending angles were quantified by Liu *et al*. (2015) using end-to-end simulations; both a clear negative tendency and an increase in the magnitude of the bending angle RIEs with solar activity were identified^9^. This study also noted that the maximum RIEs appeared at low latitudes during daytime and fell within a range of −0.03 μrad to −0.05 μrad. Qu *et al*. (2015) discussed the characteristics of second-order RIEs in radio occultation; they concluded that second-order RIEs vary with the radio occultation azimuth in a sinusoidal pattern^10^. Healy and Culverwell (2015) proposed a modification to the standard ionospheric correction method by adding a new term, κ(*a*) × (*α*_1_(*a*) − *α*_2_(*a*))^2^, to the correction function, where *a* is the impact parameter, and (*α*_1_, *α*_2_) are the L1 and L2 bending angles, respectively. The variable κ is a weak function of the impact parameter and depends on a priori ionospheric information^11^. This new correction model provides a feasible means of mitigating the RIEs, and it is worthwhile and necessary to characterize the key parameter κ.

In this paper, an idealized model that employs empirical atmospheric and ionospheric models is proposed to simulate the RO bending angles and the RIEs. The simulation schemes for different latitudes and local times are used to investigate the latitudinal and diurnal variations in RIEs and the key parameter, κ, which is applied in a new ionospheric correction method. A characterization of this new parameter and its effects on bending angle retrievals is presented for the first time.

## Method of Simulating Residual Ionospheric Errors

In this study, a simple and idealized model is built to simulate the residual ionospheric errors in radio occultation. The assumption of local spherical symmetry is made in the simulation. First, the neutral atmosphere empirical model, which extends from the surface to the lower exosphere (NRLMSISE-00)^12^, and the quick ionosphere calculation model (Nequick)^13^ are used to represent the neutral atmosphere and the ionosphere, respectively. We can then obtain the neutral atmospheric refractivity and the ionospheric refractivity using the following formulas^14^:

1 $${\mu }_{{\rm{neu}}}=1+{k}_{1}\frac{P}{T}+{k}_{2}\frac{{P}_{w}}{{T}^{2}}+{k}_{3}\frac{{P}_{w}}{T},$$

2 $${\mu }_{{\rm{ion}}}=1-40.3\frac{Ne}{{f}^{2}},$$

where *μ*_neu_ represents the neutral atmospheric refractive index; *μ*_ion_ represents the ionospheric refractive index; the three constants, *k*_1_, *k*_2_ and *k*_3_, are equal to 77.6 × 10^−6^, 0.3739, and 70.4 × 10^−6^, respectively; *P* is the dry atmospheric pressure; *P*_*w*_ is the water vapor pressure; *T* is the atmospheric temperature; *Ne* is the electron density; and *f* is the radio wave frequency. The frequencies of GPS signals L1 and L2 are *f*_1_ = 1.57542 GHz and *f*_2_ = 1.22760 GHz, respectively. The simulation of the bending angle RIEs focuses on the heights above 20 km, where the effects of water vapor are negligible. Thus, the neutral atmospheric refractive index, which depends only on atmospheric pressure and temperature, can be expressed as:

3 $${\mu }_{{\rm{neu}}}=1+77.6\times {10}^{-6}\frac{P}{T}.$$

Two schemes are compared to investigate the bending angle RIEs: (1) simulation of the non-ionospheric bending angles using NRLMSISE-00 only and (2) simulation of the bending angles using both the atmospheric and ionospheric models. The atmospheric refractive index without the ionosphere is equal to *μ*_neu_, and the atmospheric refractive index including the ionosphere is given by

4 $${\mu }_{\mathrm{neu}\_\mathrm{ion}}=1+77.6\times {10}^{-6}\frac{P}{T}-40.3\frac{Ne}{{f}^{2}}.$$

**Figure 1:**
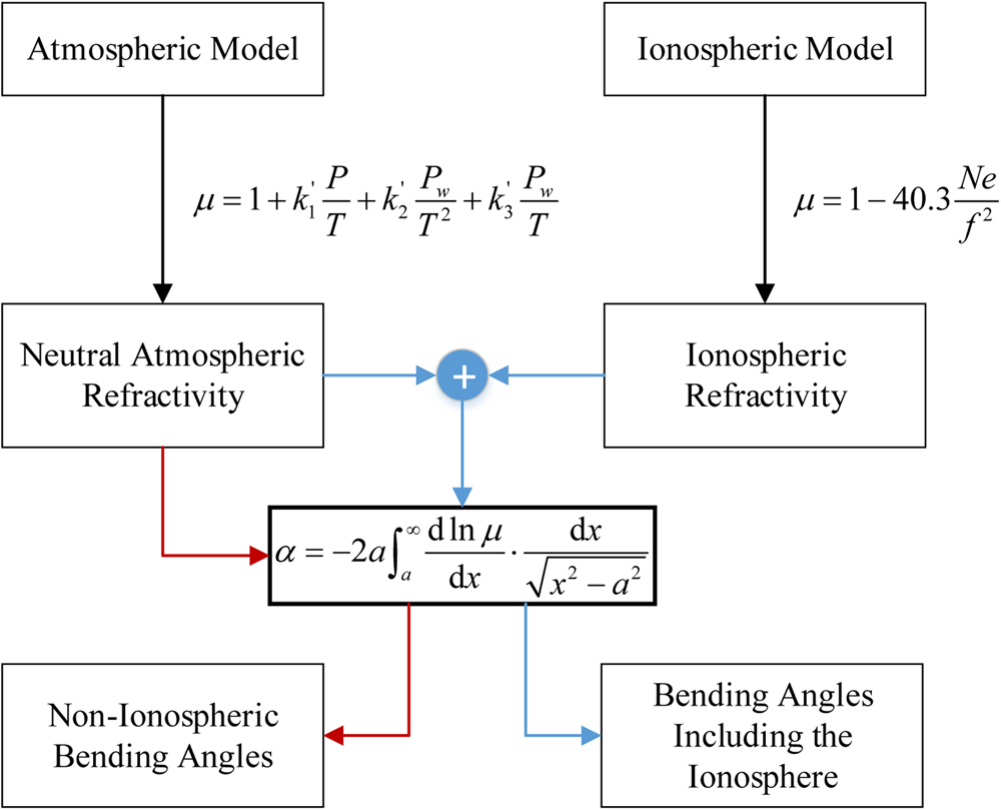
Flow chart of the process used in simulating the bending angles. This figure was produced using Microsoft Visio 2013 (http://www.microsoft.com/).

Afterwards, the bending angles can be calculated from the refractive index via conversion using Abel integration. Thus, the non-ionosphere bending angles *α*_N_ can be obtained from *μ*_neu_, and the bending angles *α*_1_ and *α*_2_ including the ionosphere (which correspond to the L1 and L2 GPS signals, respectively) can be obtained from *μ*_neu_ion_. Figure 1 shows a detailed flow chart of the process used in calculating the bending angles.

The dual-frequency linear combination of bending angles retrieved from the two frequencies at a common impact parameter proposed by Vorob’ev and Krasil’nikova (1994) is recognized as the standard ionospheric correction method that is employed in the radio occultation retrieval process. The corrected neutral atmospheric bending angle *α*_c_ at an impact parameter *a* is estimated as

5 $${\alpha }_{{\rm{c}}}(a)=({{f}_{1}}^{2}{\alpha }_{1}(a)-{{f}_{2}}^{2}{\alpha }_{2}(a))/({{f}_{1}}^{2}-{{f}_{2}}^{2}).$$

This approach is effective in removing the first-order ionospheric contributions; however, the higher-order ionospheric residuals remain, and the remaining RIE can be estimated with *RIE* = *α*_N_ − *α*_c_, where *RIE* represents the residual ionospheric error. To mitigate the residual ionospheric error, Healy and Culverwell (2015) proposed a modification to the standard ionospheric correction of the form

6 $${\alpha }_{{\rm{c}}}\text{'}(a)=({{f}_{1}}^{2}{\alpha }_{1}(a)-{{f}_{2}}^{2}{\alpha }_{2}(a))/({{f}_{1}}^{2}-{{f}_{2}}^{2})+{\rm{\kappa }}(a){({\alpha }_{1}(a)-{\alpha }_{2}(a))}^{2},$$

where the new term, κ(*a*) × (*α*_1_(*a*) − *α*_2_(*a*))^2^, compensates for the systematic residual errors of the standard ionospheric correction. Danzer *et al*.^15^ assessed this new RIE model and demonstrated that the model performs well in studies of GPS radio occultation^15^. The variable κ(*a*) is a key parameter in the RIE model, and it can be estimated as

7 $${\rm{\kappa }}(a)=RIE/{({\alpha }_{1}(a)-{\alpha }_{2}(a))}^{2}.$$

The parameter, κ, is defined as a constant by Danzer *et al*.^15^; however, there is evidence, as shown in Healy and Culverwell (2015), that κ decreases with height. In this paper, the parameter κ is estimated as κ(*a*) = **A** − B*(*a* − 20)/60, where **A** and **B** are two constants that indicate the value of κ at 20 km and the variation in the value of κ between 20 km and 80 km, respectively.

## Results of Testing the Model

**Figure 2:**
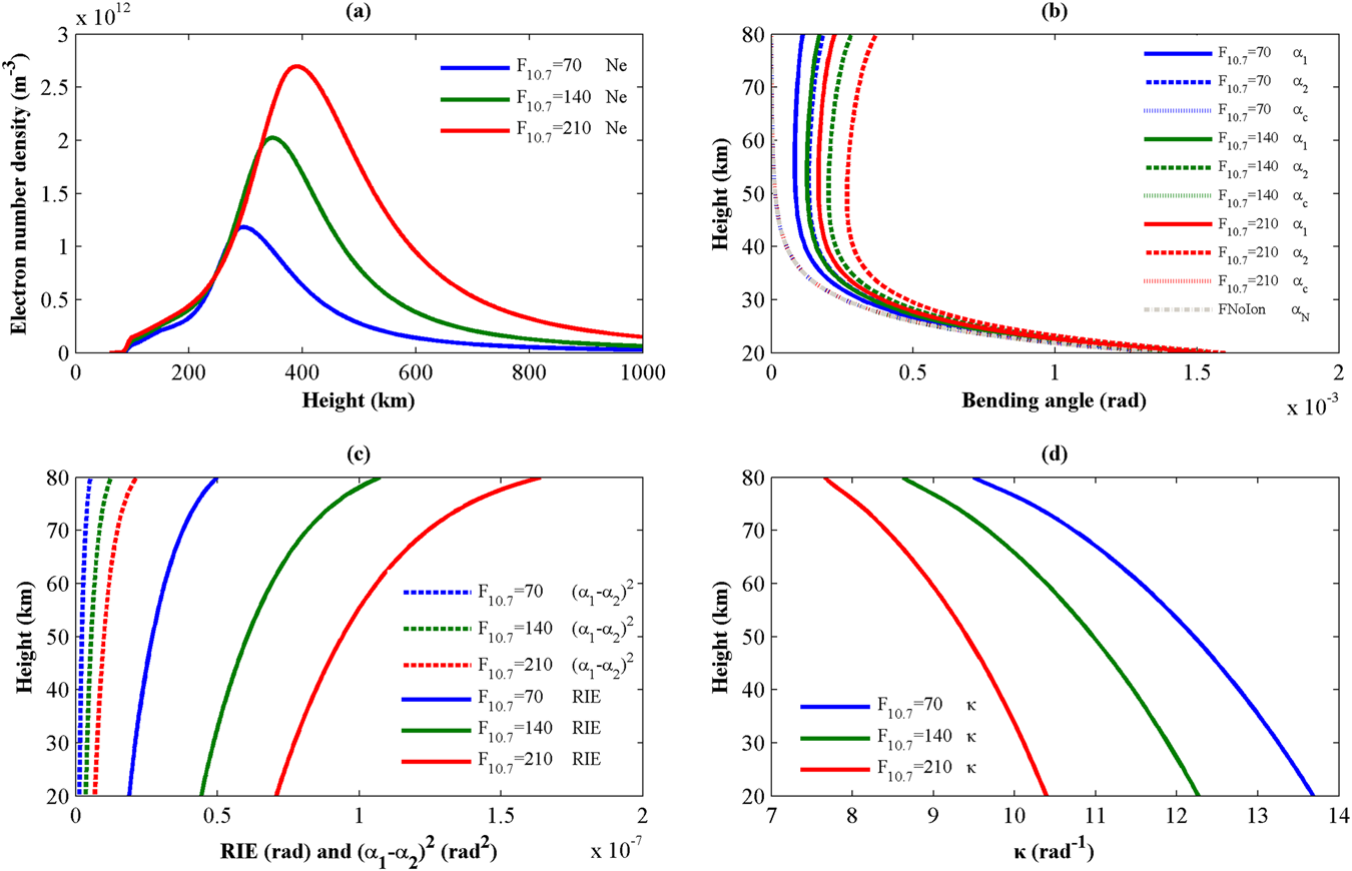
Comparison of the electron number density (**a**) and the bending angle (**b**), the bending angle RIE and (α1 − α2)^2^ (**c**), and κ (**d**) for different solar activity levels. These plots were created using MATLAB R2010b (http://www.mathworks.com/).

In this section, the proposed model for simulating the residual ionospheric error in radio occultation is tested. For this test, the site is located at a latitude of 20°N and a longitude of 120°E, and the test is conducted during the month of December at a local time of 12 o’clock. The heights used in the atmospheric model and the ionospheric model are in the ranges of 20–120 km and 60–1000 km, respectively. Different solar activity levels of F_10.7_ = 70, 140, and 210 are used in the atmospheric and ionospheric models to study the relationship between solar activity and the residual ionospheric error. Figure 2 shows the results simulated by the proposed model; in the legend of Fig. 2(b), FNoIon indicates that the bending angles are simulated without consideration of the ionosphere.

The ionospheric electron number densities are presented in Fig. 2(a). The electron number density profiles clearly vary with the solar activity level, and the peaks increase with the solar activity level. Figure 2(b) presents the L1, L2 and the ionosphere-corrected bending angles, as well as the bending angles simulated without considering the ionosphere, for the three solar activity levels. From (b), we see that the ionosphere-corrected bending angles for the different solar activity levels are consistent with the neutral atmospheric bending angles, which are simulated without consideration of the effects of the ionosphere. The standard ionosphere correction method is effective in extracting the neutral atmospheric information from the L1 and L2 bending angles. On the other hand, the residual ionospheric errors shown in Fig. 2(c) are still appreciable and have a magnitude of 10^−7^, and the RIEs increase with increasing height and increasing ionospheric electron density. Several scholars have found that adding a bending angle bias of 0.05 μrad to an entire bending angle results in a difference of about 0.5 K at an altitude of 30 km^16^. For comparison, RIEs with a magnitude of 10^−7^ cannot be neglected in high-accuracy applications of radio occultation products. Also, the profiles of (*α*_1_ − *α*_2_)^2^ are presented in Fig. 2(c); these profiles reflect magnitudes of approximately 10^−8^ and follow trends that are similar to those of the RIEs. Figure 2(d) gives insight into how κ(*a*) scales with the proposed ionospheric parameters. The value of κ decreases slowly as height increases; it is 13.8 rad^−1^ at 20 km and falls to 9.7 rad^−1^ at 80 km at a solar activity level F_10.7_ = 210. Another noteworthy phenomenon is that the κ values decline as the ionospheric electron densities increase because, in addition the variations in ionospheric electron density, the relative variations in the values of (*α*_1_ − *α*_2_)^2^ are greater than those of the RIEs. The inverse relationship between κ and the ionospheric thickness proposed by Healy and Culverwell (2015) provides a good description of the variations in κ shown in Fig. 2(d).

**Figure 3:**
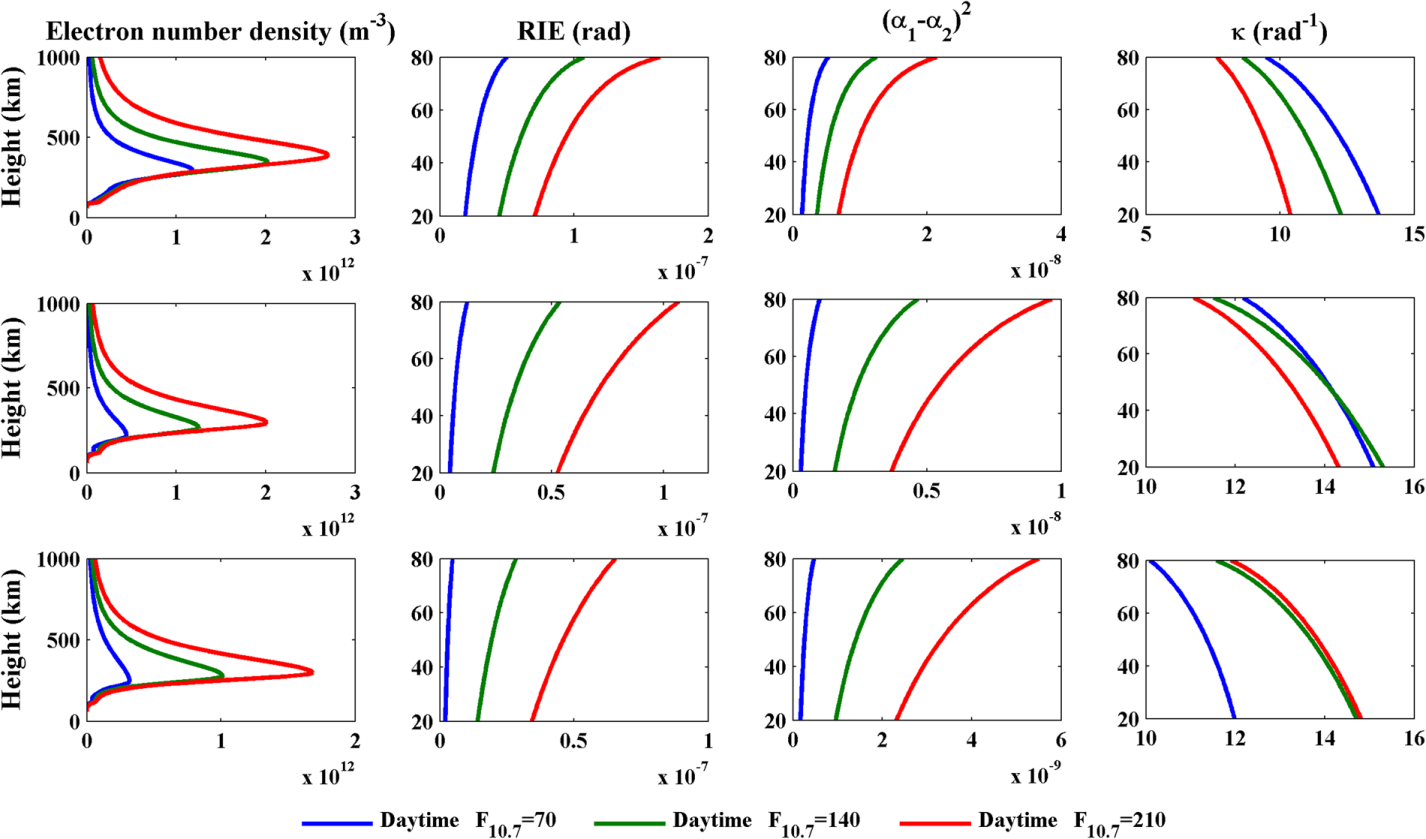
Daytime profiles of the electron number density (first column), the RIE (second column), (α1 − α2)^2^ (third column) and κ (fourth column) for latitudes of 20°N (first row), 40°N (second row) and 60°N (third row). These plots were created using MATLAB R2010b (http://www.mathworks.com/).

**Figure 4:**
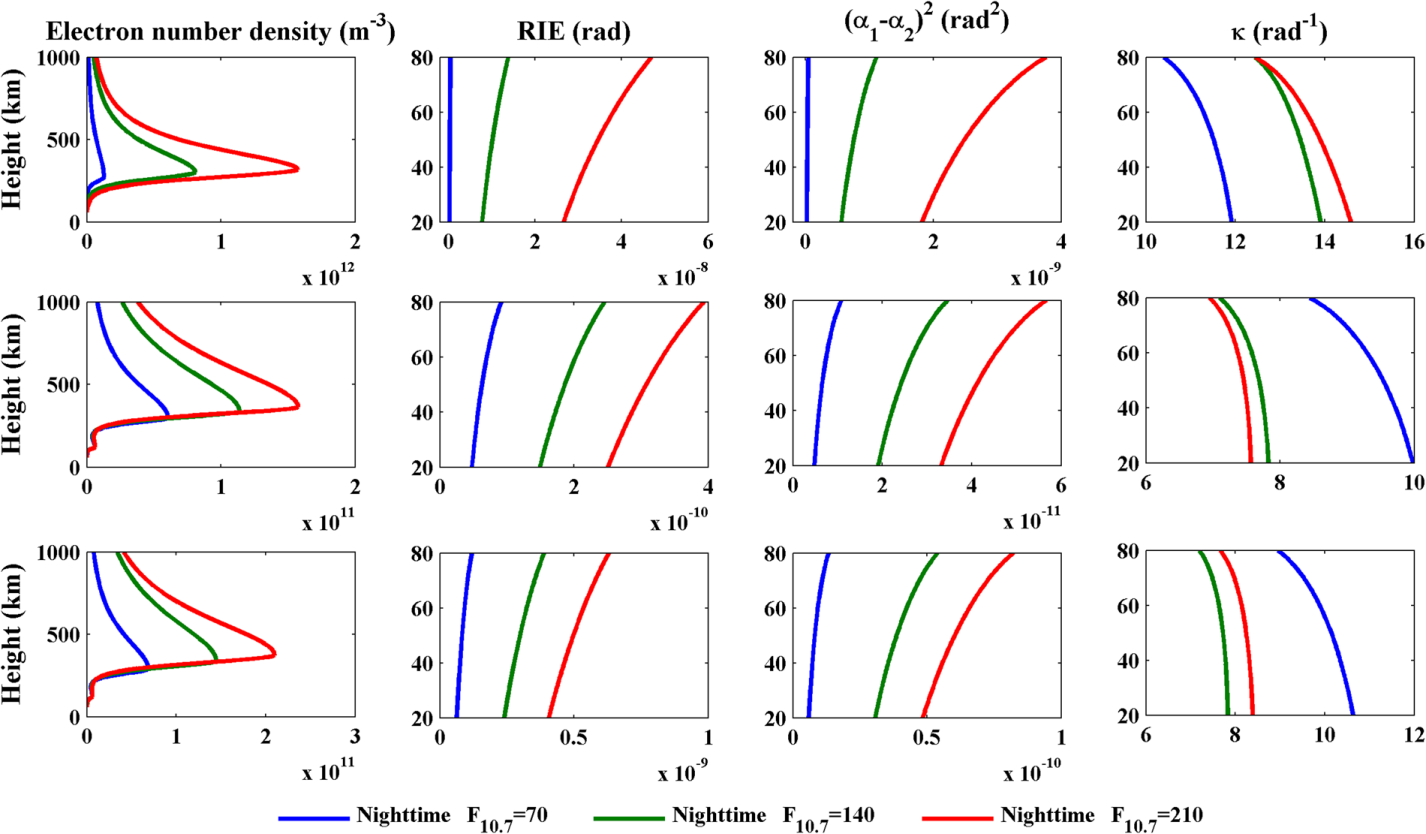
As Fig. 3, but at night. These plots were created using MATLAB R2010b (http://www.mathworks.com/).

On the basis of the simulation scheme above, new tests in which the latitude is changed to 40°N or 60°N and the local time is changed to 00:00 are performed to investigate the latitudinal and diurnal variations in the RIEs and the values of κ. Figures 3 and 4 show all of the simulated results for different latitudes, different solar activity levels, and during the day and at night, respectively. In the legend, “Daytime” and “Nighttime” represent 12:00 local time and 00:00 local time, respectively.

**Figure 5:**
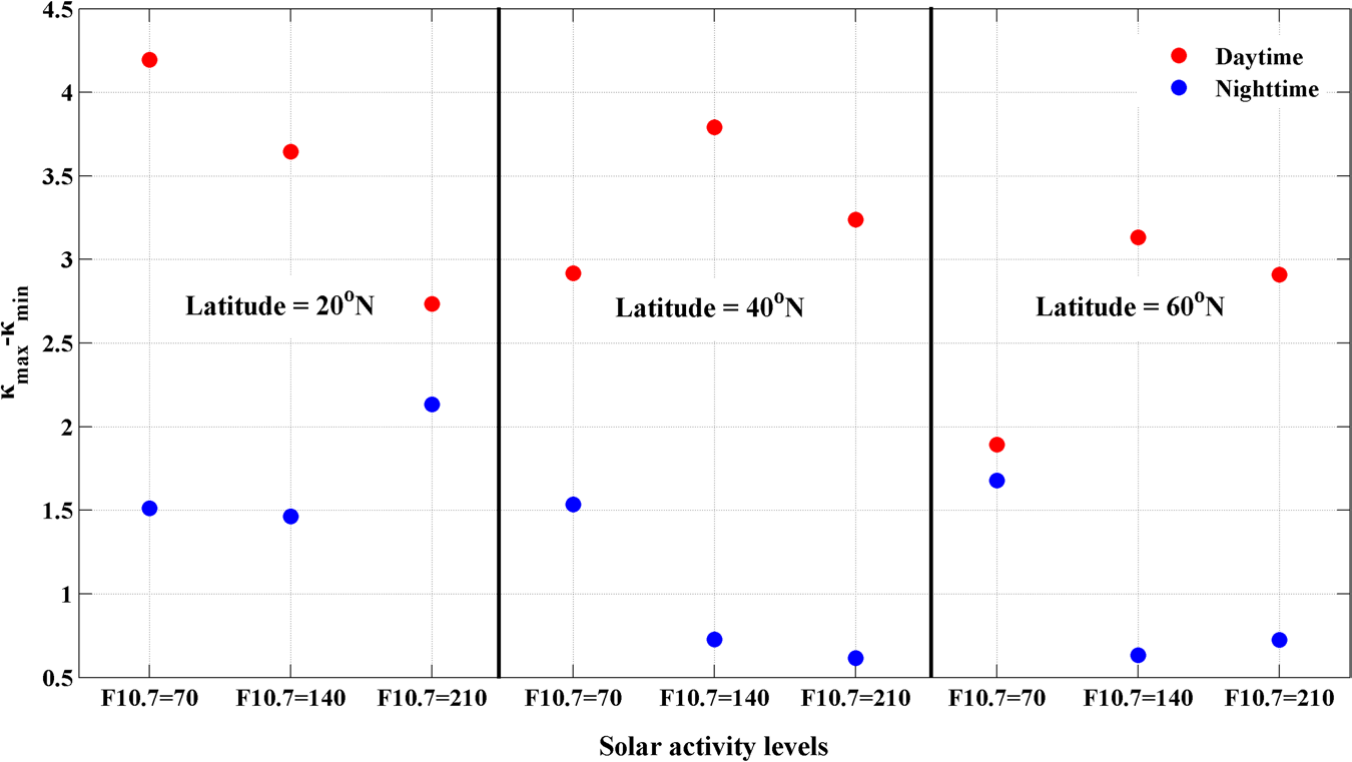
A scatter diagram of κmax-κmin values for all of the cases simulated. These plots were created using MATLAB R2010b (http://www.mathworks.com/).

The first column of Fig. 3 and the first column of Fig. 4 show that the electron number density during the day is obviously greater than that at night, and it tends to decrease with increasing latitude. The RIEs presented in the second column and the (α_1_ − α_2_)^2^ values presented in the third column show the same latitudinal and diurnal variations with the ionospheric electron number density. On the other hand, the variations in the κ values presented in the fourth column of Figs 3 and 4 show different characteristics than the previous three parameters. The values of κ at all latitudes and both during the day and at night fall within a range of 7~15 rad^−1^, and they generally decrease with increasing height. At middle and high latitudes, the κ values during the daytime are greater than those at night. However, the diurnal variations of the values of κ at low latitudes vary with the solar activity level. The κ values at night are greater than those that occur during the day when F_10.7_ = 140 or 210; however, when F_10.7_ = 70, the daytime κ profile crosses the nighttime profile. A general law that is not obvious but does exist states that the slopes of the κ profiles during the day tend to be greater than those that occur at night. This law can be easily identified from Fig. 5, which presents a scatter diagram of κ_max _− κ_min_ values for all of the cases simulated; here, κ_max_ and κ_min_ represent the maximum and the minimum κ values, which occur at 20 and 80 km, respectively. On the whole, the RIEs and κ values are most closely related to the electron densities; the RIEs are proportional to the ionospheric electron densities, whereas there is no linear relationship between the value of κ and the ionospheric electron density. The determination of κ values would require prior ionospheric information.

**Figure 6:**
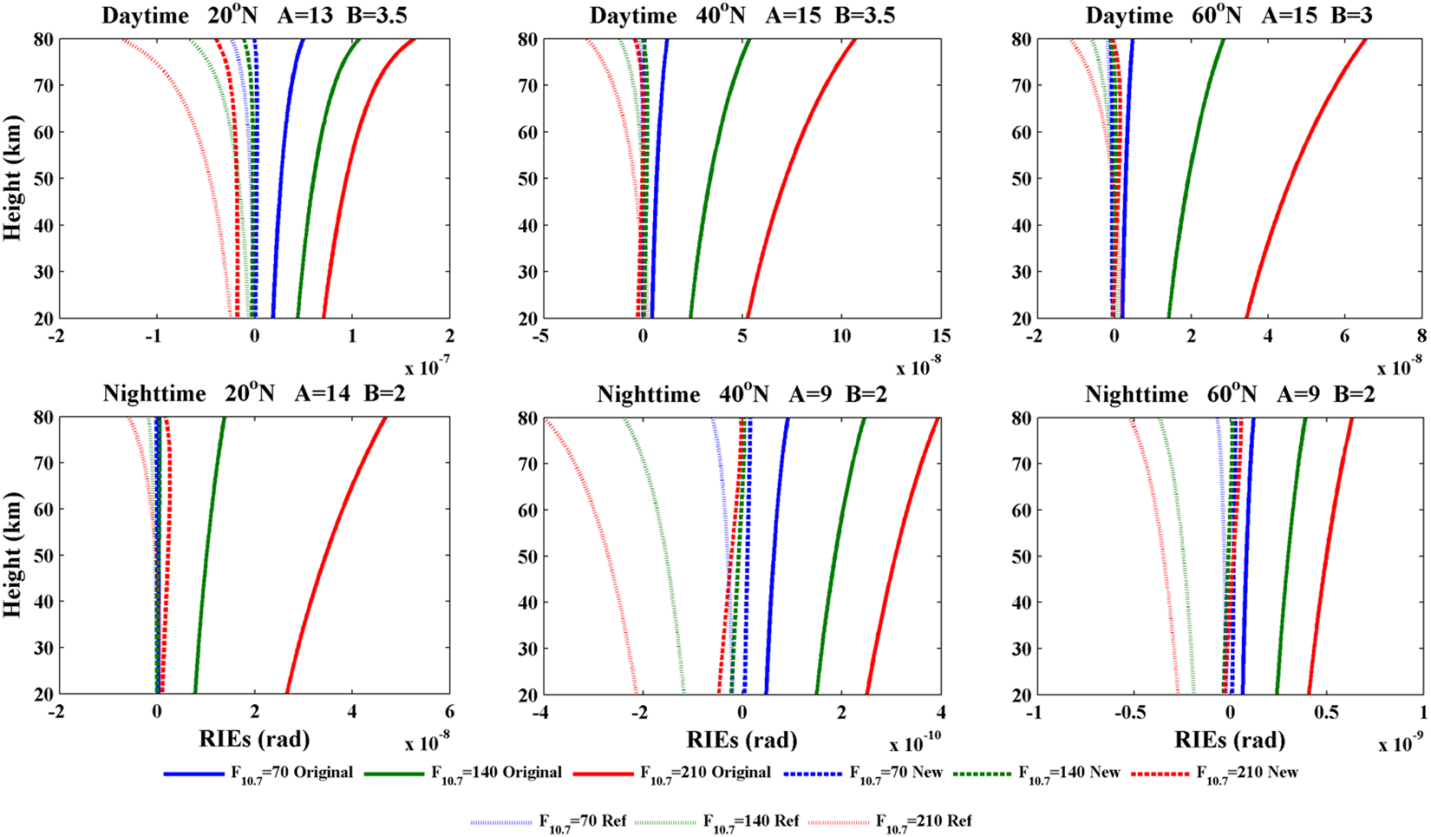
Profiles of the original RIEs, the new RIEs, and the reference RIEs at different latitudes during the day (first row) and at night (second row). Solid lines, dashed lines and dotted lines represent the original RIEs, the new RIEs and the reference RIEs, respectively. These plots were created using MATLAB R2010b (http://www.mathworks.com/).

Based on the studies above, the κ values can be simply estimated using the relationship κ(*a*) = **A** − **B** × (*a* − 20)/60. Here, **A** and **B** are constants that indicate the magnitude and the variation in the κ values, respectively, and *a* represents the impact height. The constant **B** can be estimated from the κ_max_-κ_min_ values shown in Fig. 5. The new residual ionospheric errors can be estimated as α_N_(*a*)-α′_c_(*a*) when the κ values are applied in formula (6). A comparison of the original RIEs and the new RIEs are presented in Fig. 6. The title of each panel in this figure provides information on the latitude and local time for each test, as well as the values of **A** and **B**. Also, a simple κ = 14 approximation is presented in Fig. 6, and it acts as a reference to test the new model. Figure 6 shows clearly that the new RIEs, which are calculated using appropriate **A** and **B** values estimated from Figs 3, 4 and 5, are much smaller than the original RIEs. For example, the original RIEs are approximately 5 × 10^−8^ rad at a latitude of 40°N during the day and at a solar activity level of F_10.7_ = 210; on the other hand, the new RIEs are smaller and have a magnitude of 1 × 10^−9^ rad. Comparison with the reference RIEs, which are simply calculated using κ = 14, shows that the new model displays some improvement, especially at 40°N and 60°N at night. On the other hand, the original RIEs at 40°N and 60°N at night, as shown in Fig. 4, are very small and have magnitudes of ~10^−10^; moreover, the impact of the choice of κ is less pronounced. Above all, the application of κ values in ionospheric correction substantially reduces the residual ionospheric errors, and the new model that incorporates κ behaves better than the simple relationship with κ = 14.

## Conclusion and Discussion

An idealized model that is based on the NRLMSISE-00 atmospheric model and the Nequick ionospheric model is proposed to simulate the residual ionospheric error in radio occultation. An example is first used to test this model, and the results demonstrate that the simulation model is effective and appropriate. The simulation results reveal that the dual-frequency linear combination of the L1 and L2 bending angles can remove the major ionospheric contributions. Nevertheless, small ionospheric residuals remain, and their magnitude is approximately 10^−7^. These residuals can lead to significant errors in radio occultation products, especially at heights above 30 km. The residual ionospheric errors generally increase with height and ionospheric electron density. Another important feature of RIEs is a certain relationship with the values of (*α*_1_ − *α*_2_)^2^. The κ values, which result from dividing the RIEs by the (*α*_1_ − *α*_2_)^2^ values, fall within a range of 7~15 rad^−1^ and decrease slowly with height. Afterwards, more simulation schemes for different latitudes and different local times are proposed to enable study of the latitudinal and diurnal variations of the RIEs and the values of κ. Together with the ionospheric electron density, the RIEs decrease with increasing latitude, and these quantities are greater during the day than at night. The simulation results agree with those of Angling *et al*.^17^, who modelled κ while considering the effects of geophysical parameters (e.g., the solar zenith angle, solar flux and altitude). However, there is no universal law that describes the latitudinal or diurnal variations in the value of κ; thus, the determination of κ values requires a priori ionospheric information.

Due to its remarkable advantages compared to traditional measuring techniques, the radio occultation technique is currently widely used to observe the atmosphere for global climate monitoring; however, radio occultation products do not fully meet the required precision for monitoring long-term trends in the Earth’s climate. The residual ionospheric error is one of the main sources of error in radio occultation, especially at heights above 30 km. Healy and Culverwell (2015) proposed a new term to mitigate the RIEs, which is verified in this paper. With a priori ionospheric information, it is possible to largely eliminate the residual ionospheric errors using the relationship between the RIEs and the L1 and L2 bending angles. In summary, the proposed simulation model is effective, and the results show that the relationship between the RIEs and the L1 and L2 bending angles deserves more attention.

### Data Availability

The datasets generated and analysed during the current study are available from the corresponding author upon reasonable request.
